# Combining deep learning with a kinetic model to predict dynamic PET images and generate parametric images

**DOI:** 10.1186/s40658-023-00579-y

**Published:** 2023-10-24

**Authors:** Ganglin Liang, Jinpeng Zhou, Zixiang Chen, Liwen Wan, Xieraili Wumener, Yarong Zhang, Dong Liang, Ying Liang, Zhanli Hu

**Affiliations:** 1 Lauterbur Research Center for Biomedical Imaging, Shenzhen Institute of Advanced Technology, Chinese Academy of Sciences, Shenzhen, 518055 China; 2 Shenzhen College of Advanced Technology, University of Chinese Academy of Sciences, Beijing, China; 3 Department of Nuclear Medicine, National Cancer Center, National Clinical Research Center for Cancer, Cancer Hospital & Shenzhen Hospital, Chinese Academy of Medical Sciences and Peking Union Medical College, Shenzhen, 518116 China

**Keywords:** Parametric imaging, Image generation, Deep learning, Kinetic model, Dynamic PET images

## Abstract

**Background:**

Dynamic positron emission tomography (PET) images are useful in clinical practice because they can be used to calculate the metabolic parameters (*K*_*i*_) of tissues using graphical methods (such as Patlak plots). *K*_*i*_ is more stable than the standard uptake value and has a good reference value for clinical diagnosis. However, the long scanning time required for obtaining dynamic PET images, usually an hour, makes this method less useful in some ways. There is a tradeoff between the scan durations and the signal-to-noise ratios (SNRs) of *K*_*i*_ images. The purpose of our study is to obtain approximately the same image as that produced by scanning for one hour in just half an hour, improving the SNRs of images obtained by scanning for 30 min and reducing the necessary 1-h scanning time for acquiring dynamic PET images.

**Methods:**

In this paper, we use U-Net as a feature extractor to obtain feature vectors with a priori knowledge about the image structure of interest and then utilize a parameter generator to obtain five parameters for a two-tissue, three-compartment model and generate a time activity curve (TAC), which will become close to the original 1-h TAC through training. The above-generated dynamic PET image finally obtains the *K*_*i*_ parameter image.

**Results:**

A quantitative analysis showed that the network-generated *K*_*i*_ parameter maps improved the structural similarity index measure and peak SNR by averages of 2.27% and 7.04%, respectively, and decreased the root mean square error (RMSE) by 16.3% compared to those generated with a scan time of 30 min.

**Conclusions:**

The proposed method is feasible, and satisfactory PET quantification accuracy can be achieved using the proposed deep learning method. Further clinical validation is needed before implementing this approach in routine clinical applications.

## Background

When positron emission tomography (PET) was first proposed, it showed good contrast for performing target area imaging with high quality [1]. As research into fluorodeoxyglucose (FDG) has deepened, the innocuous nature of FDG and its high tumor uptake percentage compared to that of other tissues have allowed PET imaging to show strong tumor diagnosis potential [2]. Chemotherapy and chemoradiotherapy patients are increasingly monitored using PET with ^18^F-FDG [3]. In routine clinical practice, the standard uptake value (SUV) is highly applied because the glucose metabolic rate and SUV have a good relationship, and this index is easy to obtain [4]. However, the SUVs of static PET images are affected by many different factors, such as the variable uptake period (the time between injection and imaging) and reconstruction parameters (filters, number of iterations, and decay correction) of different scanning instruments, making it problematic to compare SUVs acquired in different places. For this reason, graphical methods such as the Patlak plot are more promising due to their robustness and simplicity in clinical use case [5]. When rigorous and reliable, quantitative analyses can offer more valuable information for clinical practice [6].

Dynamic PET images form a better imaging modality for calculating quantitative values. The first few frames of a dynamic PET image are very short, resulting in considerable noise and a low signal-to-noise ratio (SNR) [7]. Therefore, in most cases, the scanning time required for each time frame of dynamic PET images gradually increases, and the whole process takes at least an hour so that the time activity curve (TAC) is highly accurate. Many parameters can be computed once the TAC is obtained. With the Patlak plot method, the *K*_*i*_ parameter, which is the net uptake rate constant, is used most often. PET Patlak parametric images have been generated based on direct reconstruction using different methods (e.g., the kernel method [8–10], deep image prior with the alternating direction of multipliers method (ADMM) [11–14], the hybrid approach [15], and a method with only a deep network [16]). These methods make the reconstruction process much longer when obtaining parametric images, and some methods do not work well for real patient data due to the fact that they conduct training with simulated data. Therefore, none of these methods can be used in clinical practice. Parametric imaging is time-consuming, and the resulting noisy images require interpretation by skilled users [17]. By reducing the image noise and generation time, parametric images can be made available for clinical use much more quickly. In our study, we made the first attempt to solve this problem. We used only the first 30 min of dynamic PET images. After applying our algorithm, we obtained higher-quality parametric images than those acquired after scanning for 30 min, thus reducing the original 1-h scanning time to half an hour.

## Methods

### Feature extraction network

**Fig. 1:**
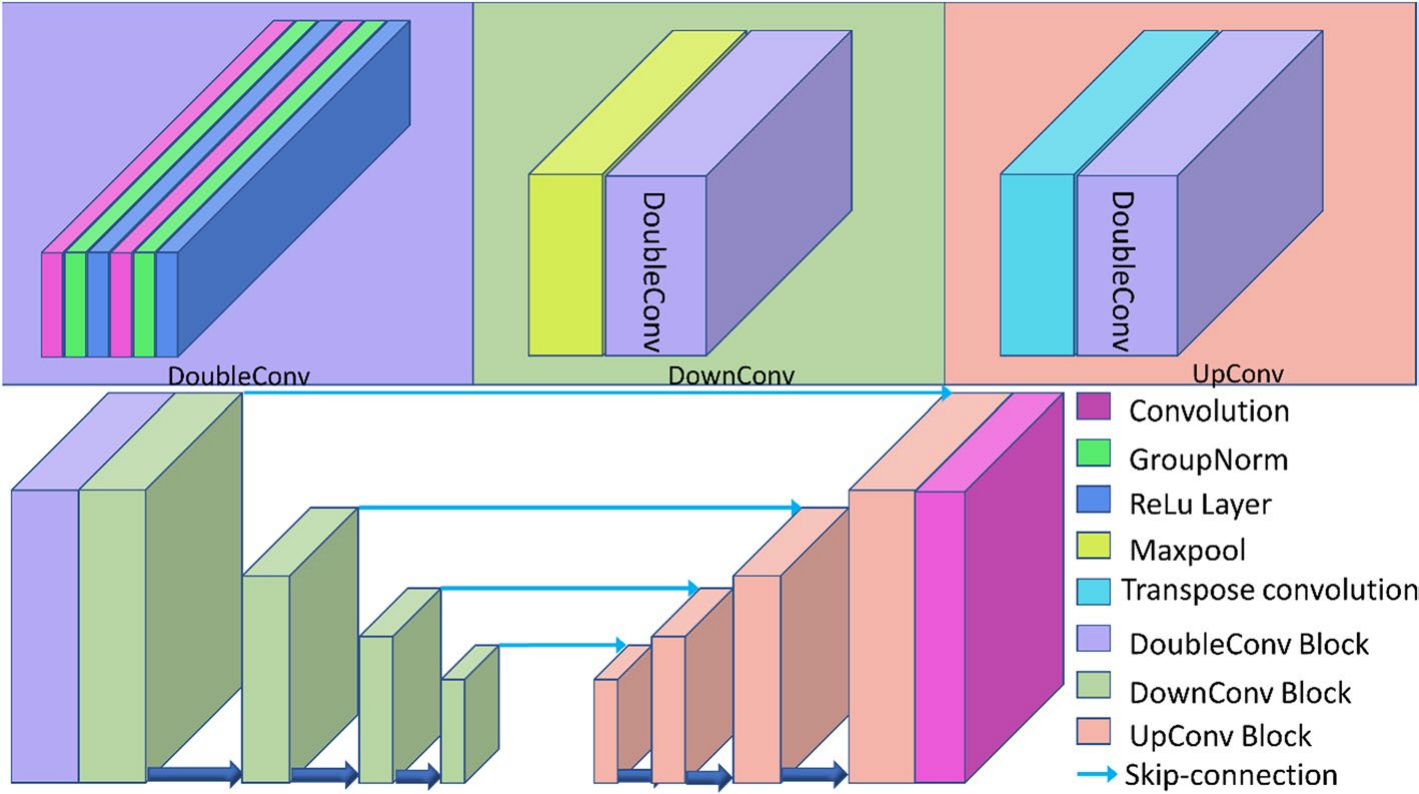
The architecture of the feature extraction network. To demonstrate the effectiveness of our approach, we did not make many structural improvements to U-Net

**Fig. 2:**
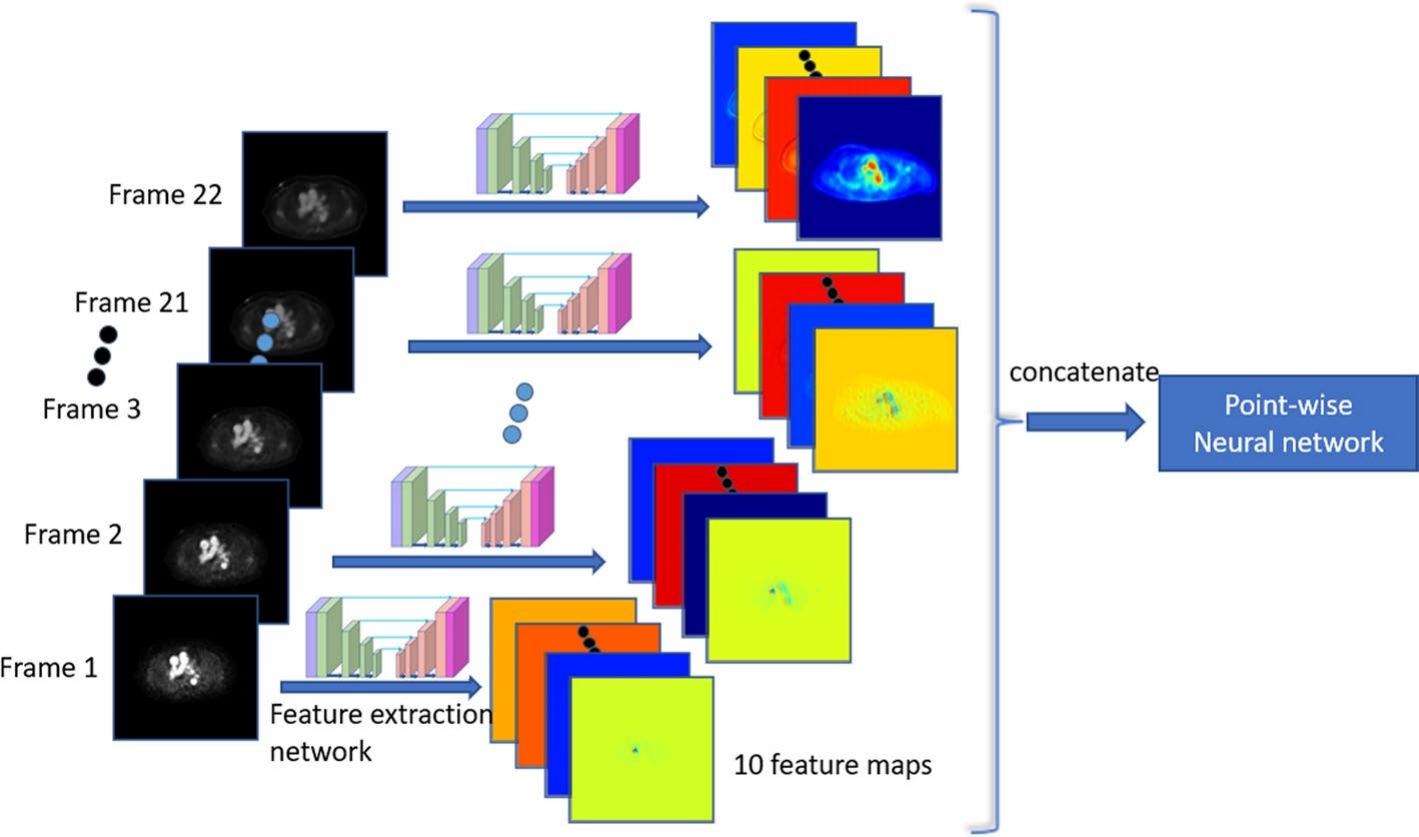
The relation between the feature extraction network and the kinetic model network (pointwise neural network). We obtained each frame’s feature maps using U-Net. All 220 feature maps were then input into a pointwise neural network, which was implemented by a stack of convolutions with a kernel size of 1. Therefore, each voxel was a 220-dim vector that was fed into the kinetic model network

**Fig. 3:**
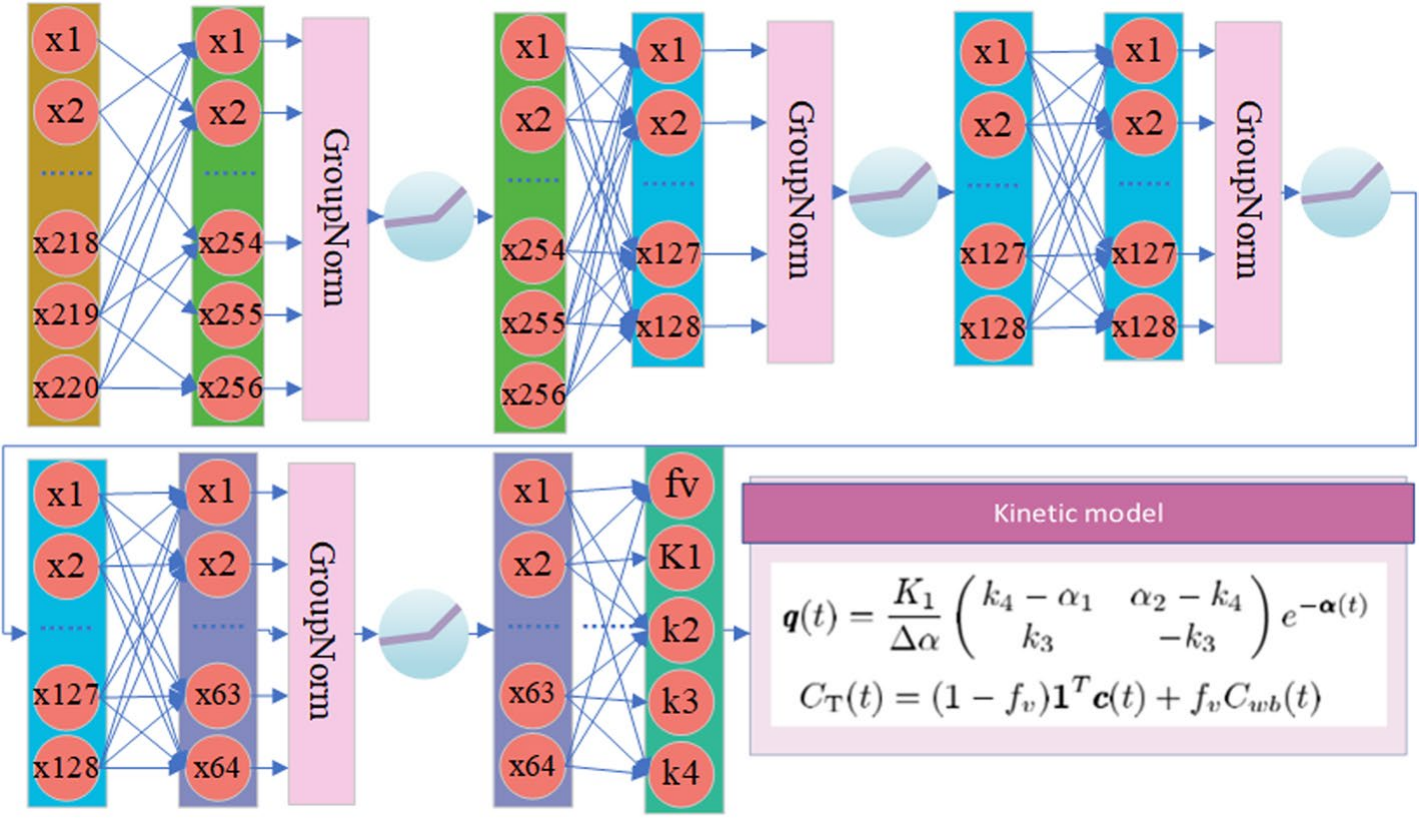
The architecture of the kinetic model network. Each rectangle with different colors surrounding a group of neurons represents a hidden layer with different numbers of neurons, and layers with the same color possess the same number of neurons

In computer vision, an increasing amount of research points to the importance of convolutional neural networks. Properly trained convolutional neural networks have superior effects in image generation, image segmentation, and other aspects that surpass those of traditional computer vision-based processing methods. At the same time, convolutional neural networks can automatically extract the features of images through training. Based on these studies, we build a fully convolutional neural network with a network architecture that looks like the U-Net architecture that is often used in medical image segmentation. An encoder first downsamples the original 1-channel SUV images. Then, the high-level semantic information of the image is encoded through a series of convolutional or pooling operations to obtain an image feature vector. This feature vector is then sent to a decoder, which returns the information the encoder takes out. This process eliminates noise, which is harder to learn or fit into the network than a useful signal. We change the batch normalization operation in the network to a group normalization operation and add skip connections, similar to those in the residual network, to speed up the training process and improve the quality of the generated images. To be more specific, a basic block called a DoubleConv block makes up many other blocks. The GroupNorm layer and the activation layer come after the two convolutional layers in the DoubleConv block. The rectified linear unit (ReLU) function is chosen as the activation function. The number of channels per group is set to 16 for GroupNorm. The encoder comprises one DoubleConv block and four DownConv blocks that are made up of one maximum pooling layer and one DoubleConv block. The Maxpool layer’s function is to perform downsampling by a factor of 2. The first DoubleConv block maps the channel size of the input image to the target channel size for the subsequent calculations. The target channel sizes of the blocks are set to 64, 128, 256, 512, and 1024, which means that the output feature image size is 1/16 of the original image size. The decoder then takes the last feature image to perform upsampling 4 times using UpConv blocks. Each UpConv block comprises one transposed convolutional layer and one DoubleConv block. For each block at the same level, skip connections are made between the encoder and decoder. The architecture of the feature extraction network is shown in Fig. 1. The dimensionality of the input is described in the “Training Setup” section, and the flow of data from one network to the other is shown in Fig. 2. In Fig. 2, we refer to the following kinetic model network as a pointwise neural network (Fig. 3).

### Kinetic model network

The physiological system of dynamic processes in the tissue of interest is decomposed into several compartments, which interact with each other. In PET, tracer kinetic modeling is based on compartmental analysis. Ordinary differential equations (ODEs) continuously and deterministically represent the compartmental system. Each equation describes the temporal rate of change exhibited by the material in a compartment. These rates of change are controlled by the physical and chemical rules that govern how materials move from one compartment to another. These rules include diffusion, temperature, and chemical reactions [18]. The vast majority of articles use the 2-tissue compartment model (2TCM) with the Patlak method to analyze dynamic PET images. Since most researchers have looked into the 2TCM and found that it works [7], our method also builds the network on the 2TCM. The ODEs of the 2TCM are described as follows:

1 $$\frac{{{\text{d}}C_{1} (t)}}{{{\text{d}}t}} = K_{1} C_{0} (t) - (k_{2} + k_{3} )C_{1} (t) + k_{4} C_{2} (t)$$

2 $$\frac{{{\text{d}}C_{2} (t)}}{{{\text{d}}t}} = k_{3} C_{1} (t) - k_{4} C_{2} (t)$$

where ${K}_{1}$ is a constant that represents the rate of influx from plasma to tissue, ${k}_{2}$ is a constant that represents the rate of efflux from the first compartment in the 2TCM, ${k}_{3}$ is the rate of transfer from a nonspecific compartment to a specific compartment in a reversible or irreversible 2TCM, and ${k}_{4}$ is the rate of transfer from a specific compartment to a nonspecific compartment in the reversible 2TCM. To increase the complexity and diversity of the TACs generated by the network, we do not fix ${k}_{4}$ as 0. However, the network is capable of generating TACs when ${\mathrm{k}}_{4}$ is equal to 0. ${C}_{0}(t)$ is the input blood function, ${C}_{1}\left(t\right)$ is the concentration of the nondisplaceable compartment, and ${C}_{2}\left(t\right)$ is the concentration of the binding radiotracer in the specific compartment; the tissue concentration ${C}_{\mathrm{T}}(t)$ is the sum of the nondisplaceable and specific compartment concentrations. [19].

The solution of these ODEs is the convolution of an exponential function with the input function. The equations are as follows:

3 $$C_{{\text{T}}} (t) = aC_{0} (t) \otimes e^{{ - \alpha_{1} t}} + bC_{0} (t) \otimes e^{{ - \alpha_{2} t}}$$

4 $$\begin{aligned} \alpha_{1} = & \left( {k_{2} + k_{3} + k_{4} - \sqrt {\left( {k_{2} + k_{3} + k_{4} } \right)^{2} - 4k_{2} k_{4} } } \right)/2 \\ \alpha_{2} = & \left( {k_{2} + k_{3} + k_{4} + \sqrt {\left( {k_{2} + k_{3} + k_{4} } \right)^{2} - 4k_{2} k_{4} } } \right)/2 \\ a = & K_{1} \left( {k_{3} + k_{4} - \alpha_{1} } \right)/\left( {\alpha_{2} - \alpha_{1} } \right) \\ b = & K_{1} \left( {\alpha_{2} - k_{3} - k_{4} } \right)/\left( {\alpha_{2} - \alpha_{1} } \right) \\ \end{aligned}$$

The total activity concentration (e.g., in nCi/ml) for a voxel at a given time is denoted by

5 $$C_{{{\text{PET}}}} \left( {\varphi_{s} ,t} \right) = \left( {1 - f_{v} } \right)C_{{\text{T}}} (t) + f_{v} C_{{{\text{WB}}}} (t)$$

where ${\mathrm{\varphi }}_{\mathrm{s}}$ represents the parameters of the kinetic model. The volume fraction of a voxel that is made up of blood is denoted by the constant ${f}_{v}$. ${C}_{\mathrm{WB}}$(nCi/ml) is the concentration of tracer activity in whole blood (i.e., plasma plus blood cells plus other particulate matter) [20].

Our method uses the blood input function $${C}_{0}\left(t\right)$$ as the whole blood function $${C}_{\mathrm{WB}}(t)$$. We form the kinetic model network, a convolutional neural network with 1 × 1 convolutional layers that let each voxel be computed separately while reducing the number of parameters and increasing the training speed of the network. We applied the feature extraction network to each individual time frame image of the dynamic PET data, extracting 10 feature maps for each time frame. With a total of 22 time frames, there are 220 feature maps in total. This means that each voxel is represented by a 220-dimensional vector, as shown in Fig. 2. The feature extraction network's output feature vectors are fed into the kinetic model network. Moreover, the kinetic model network predicts the five parameters ($${f}_{v},{K}_{1},{k}_{2},{k}_{3},{k}_{4})$$ of the 2TCM for each voxel based on the inputs. After obtaining the parameters generated by the network, we can use these parameters to obtain the whole TAC through the 2TCM. Once we have obtained the time activity curves for each voxel, we can calculate the dynamic PET image at any desired time frame. To be more specific, the intensity of the image at pixel *j* in time frame *m*, $${x}_{m}({\theta }_{j})$$, is determined by

6 $$x_{m} \left( {\theta_{j} } \right) = \mathop \smallint \limits_{{t_{m,s} }}^{{t_{m,e} }} C_{{{\text{PET}}}} \left( {\tau ;\theta_{j} } \right)e^{ - \lambda \tau } {\text{d}}\tau$$

where ${t}_{m,s}$ represents the starting time of frame *m*, ${t}_{m,e}$ represents the ending time of frame *m*, and $\lambda$ represents the decay constant of the radiotracer. ${C}_{\mathrm{PET}}\left(t;{\theta }_{j}\right)$ denotes the tracer concentration in pixel *j* at time *t*, which is determined using the aforementioned kinetic model with the parameter vector ${\theta }_{j}\in {R}^{{n}_{k}\times 1}$.

Then, we can estimate *K*_*i*_ using the Patlak plot method:

$$\frac{x(t)}{{C}_{p}(t)}={K}_{i}\frac{{\int }_{0}^{t}{C}_{p}\left(\tau \right)\mathrm{d}\tau }{{C}_{p}(t)}+{V}_{0}$$

where ${K}_{i}$ is the constant rate of irreversible binding. ${V}_{0}$ is the distribution volume of the nonspecifically bounded tracer in the tissue. $x(t)$ is the integrated activity of the tissue up to time *t*. ${C}_{p}(t)$ is the plasma concentration of the tracer at time *t*.

### Training setup

Only the dynamic images obtained during the first thirty minutes were fed into the whole neural network. All the inputs were normalized to SUV images. The input matrix had a shape of ${T}_{i}$ ×1 × *H* × *W*, where ${T}_{i}$ corresponds to the total number of time frames within the initial thirty minutes. H and W denote the height and width of the image, respectively. The number of input channels was specified as 1. The size of the output matrix, representing the whole network's output, was $T\times H\times W$, where T represents the number of time frames in the dynamic PET images. Furthermore, the loss function was the Huber loss [21], which is very resistant to outliers.

To train the kinetic model network, we calculated the loss between the generated images and the ground truth as the loss function.

7 $${\text{loss}}_{{{\text{SUV}}}} = \frac{1}{TMN}\sum\limits_{t} {\sum\limits_{x,y} {{\text{huber\_loss}}({\text{pred}}_{{{\text{SUV}}}}^{t} (x,y),gt_{{{\text{SUV}}}}^{t} (x,y))} }$$

where ${\mathrm{pred}}_{\mathrm{SUV}}^{t}(x,y)$ is the pixel value of the generated image at position (*x*,*y*) for the *t*-th frame. ${\mathrm{gt}}_{\mathrm{SUV}}^{t}(x,y)$ is the pixel value of the ground truth at position (*x*,*y*) for the t-th frame. *T* is the total number of frames. M and N are the height and width of the images, respectively. Due to the fact that the first 30 min of dynamic PET images (the first 22 frames) were already used as network inputs, we only utilized the images from the subsequent 30 min (last 6 frames) as the training targets.

Additionally, we added a time difference loss function for the linear part of the Patlak model.

8 $$\begin{aligned} y(t,x,y) \triangleq & \frac{{{\text{pred}}_{{{\text{SUV}}}} (t,x,y)}}{{C_{p} (t)}} \\ x(t) \triangleq & \frac{{\int_{0}^{t} {C_{p} (\tau ){\text{d}}\tau } }}{{C_{p} (t)}} \\ {\text{diff}}_{k} (f(t,x,y)) \triangleq & f(t_{k + 1} ,x,y) - f(t_{k} ,x,y) \\ {\text{loss}}_{{{\text{diff}}}} = & \frac{1}{{T_{{{\text{linear}}}} MN}}\sum\limits_{k} {\sum\limits_{x,y} {{\text{huber\_loss}}({\text{diff}}_{k} (y(t,x,y)),{\text{diff}}_{k} (K(x,y)x(t))} } ) \\ \end{aligned}$$

where ${C}_{p}(t)$ is the blood input function. $K(x,y)$ is the *K*_*i*_ parameter of the Patlak plot at position (*x*,*y*). *x*(*t*), *y*(*t*), and ${\mathrm{diff}}_{k}(\cdot )$ are defined according to the definitions provided in Eq. (8). ${T}_{\mathrm{linear}}$ is the total number of frames that represent the linear portion in the Patlak plot model. ${t}_{k}$ represents the *k*th time frame.

Thus, the total loss is as follows:

9 $${\text{loss}} = {\text{loss}}_{{{\text{SUV}}}} + \lambda \times {\text{loss}}_{{{\text{diff}}}}$$

where $\lambda$ is a hyperparameter that adjusts the weight between fitting *K*_*i*_ and the SUV.

We did not train the feature extraction network and the kinetic model network separately; instead, we treated them as one end-to-end network and trained them together. The optimizer was chosen as adaptive moment estimation (Adam) [22], and the learning rate was set to 1e-4. We used a strategy that adjusted the learning rate to one-tenth of the original value every 10,000 iterations, with a lower bound of 1e-7. We trained our network on an NVIDIA GeForce RTX 3090 GPU for a total of 10 epochs. Each epoch included 7171 iterations. To validate the effectiveness of our proposed method that incorporates a kinetic model, we compared it to a method with the exact same network structure but without the kinetic model. In other words, we directly predicted the SUV images for the last 30-min time frames without the need to perform the steps of the kinetic model. Additionally, while maintaining the rest of the network architecture unchanged, we removed the sigmoid activation function from the final layer. We are still employing a point-wise neural network approach. We refer to this method as "without model" in the figure. The method without incorporating the kinetic model adopted the same hyperparameter settings and loss function as the full model. This was done to minimize the influence of other factors and ensure the accuracy of the conclusions.

### Patient PET data

The network's training dataset was obtained from the Cancer Hospital of the Chinese Academy of Medical Sciences Shenzhen Center, which included 7313 slices of data from 103 patients acquired with the GE Healthcare Discovery MI Dr PET/CT Scanner. All patients had space-occupying lung lesions, which can also be called pulmonary nodules. Both benign and malignant lesions were present. We randomly selected 10 patients as the test set and 93 patients as the training set. The patient's height range was 1.641 m ± 0.089 m, and the weight range was 63.0 kg ± 10.36 kg. Information on the patient's gender and age were unavailable because the patient's data were anonymized and desensitized. The dynamic PET data were divided into 28 frames: 6 × 10 s, 4 × 30 s, 4 × 60 s, 4 × 120 s, and 10 × 300 s with total radionuclide doses of ${\mathrm{F}}^{18}$-FDG ranging from 201.83 Mbq to 406.46 Mbq for different patients. Each time frame of the dynamic PET data was an image array of 256 × 256 × 71 voxels with a voxel size of 1.95 × 1.95 × 2.79 mm3. The blood input function was manually extracted from the image region of the descending aorta.

## Results

### Qualitative image quality assessment

**Fig. 4:**
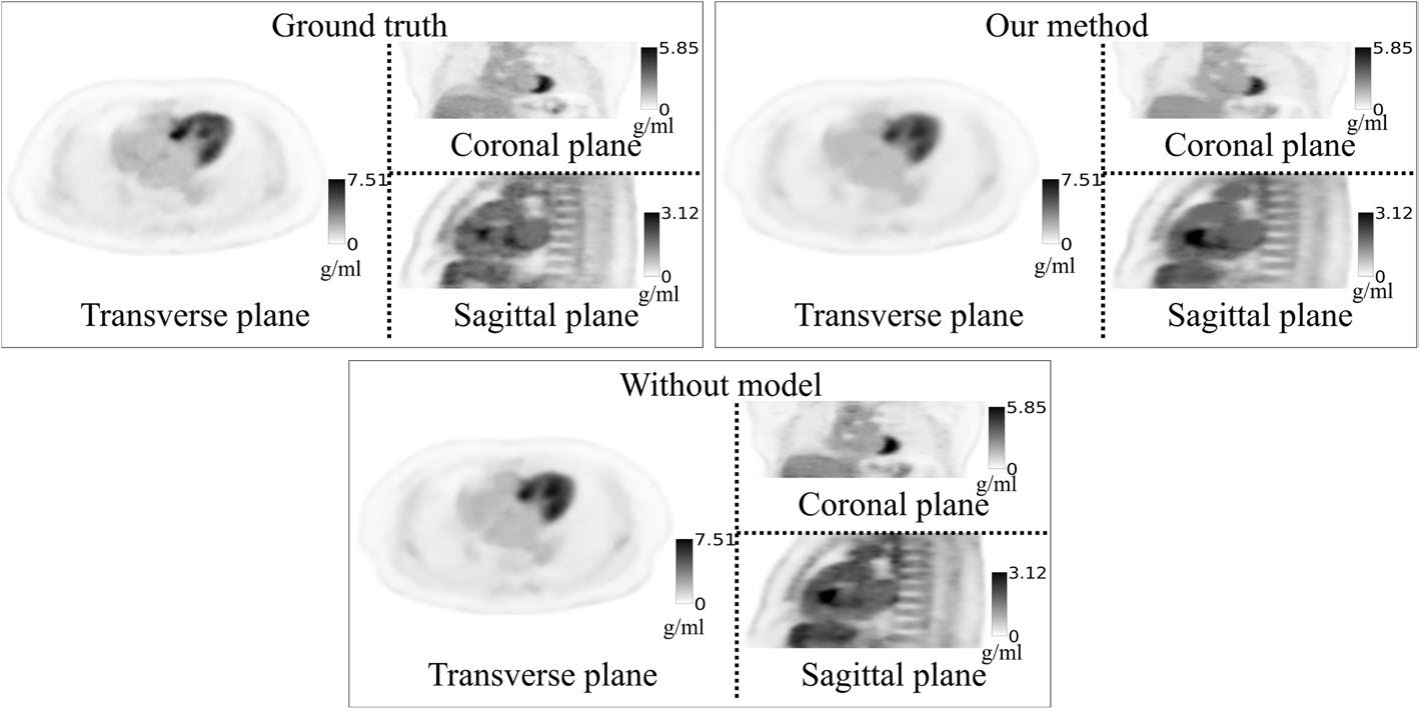
Examples of generated dynamic PET images obtained from different planes (the transverse plane, coronal plane and sagittal plane). The ground truths are the last frames of the dynamic PET images, which were obtained 1 h after injection with a 5-min scanning time. The images in the upper-right corner were obtained by our proposed network with the kinetic model, and the images on the bottom were generated by the same network without the kinetic model

**Fig. 5:**
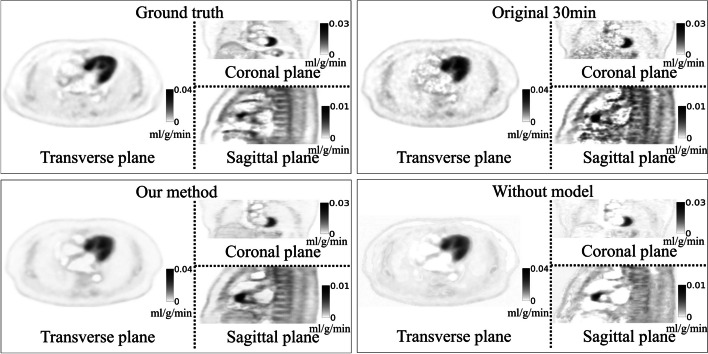
Example of generated parametric *K*_*i*_ images obtained from different planes (the transverse plane, coronal plane and sagittal plane). The *K*_*i*_ images of both the ground truth and our proposed method were obtained by fitting the last 13 frames of the Patlak plot’s data points with linear regression. The main differences between both methods are that the data points from the last 30 min were generated by the proposed network rather than being real. The *K*_*i*_ images of the method without the kinetic model in the lower right were generated directly rather than by fitting a Patlak plot. The images in the upper right were obtained by fitting only the first 30 min of frames

**Fig. 6:**
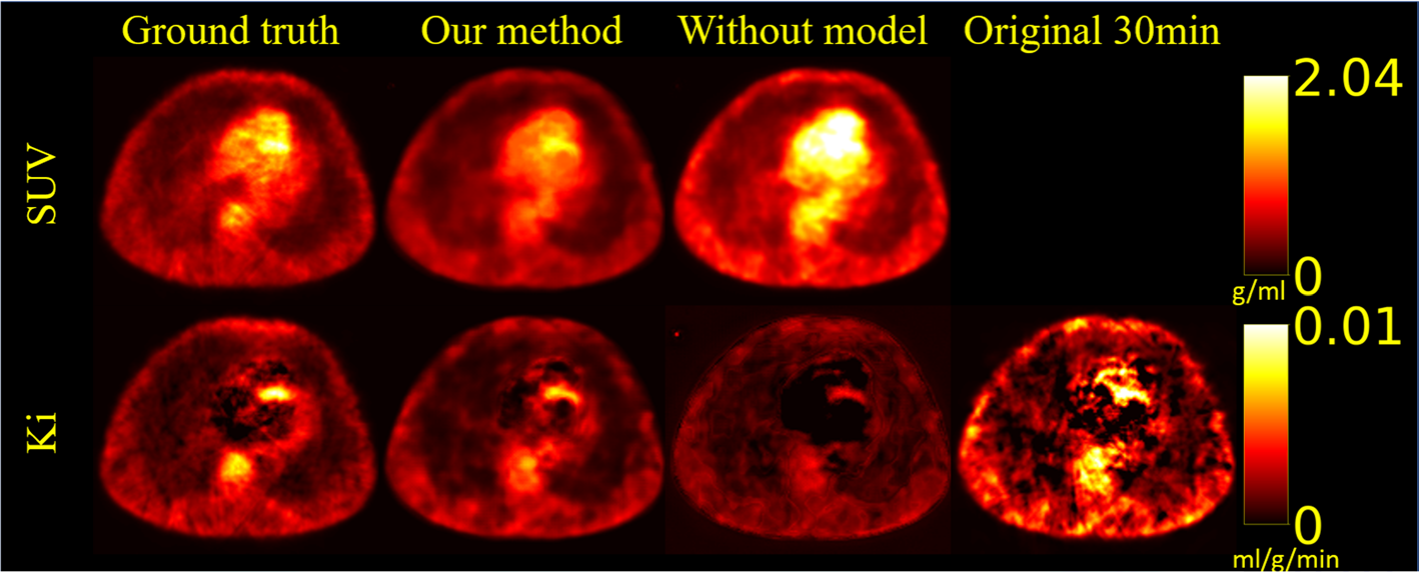
An example of a slice of an SUV image and a slice of a *K*_*i*_ image rendered in pseudocolor

Figure 4 shows that the overall visual effect of the generated images was close to that of the reference images and presented most of the anatomical structure details, which was based on the observations of the three views in the coronal, sagittal, and transverse planes. In addition, some high-uptake regions could still be effectively represented in the generated images. A better SNR could be obtained using our proposed method, which also had a positive effect in terms of noise reduction for improving the image quality. Figure 5 shows that a noisy *K*_*i*_ image would have been obtained if we applied the Patlak plot method on the first 30 min of dynamic PET data. However, we can see that the noise level was reduced through our method, and we could generate a more reasonable *K*_*i*_ image. Our method could show more anatomical details of tissues and organs than the no-kinetic-model network. Both our network and the no-kinetic-model network exhibit artifacts in the cardiac region in Figs. 4 and 5. This phenomenon is likely attributed to the fact that the network's input consists of various time frames from the initial 30 min. Due to cardiac motion between time frames, the lack of consistency in features extracted by the feature extraction network introduces significant noise, resulting in the appearance of artifacts. Figure 6 shows that our method gave more accurate SUV results in most tissue regions, but it provided SUVs that were lower than the real values in some metabolically active areas that did not fit the kinetic model. However, if we did not have kinetic models, our networks may have produced very inaccurate predictions about some tissues and organs. This would make the images less useful for diagnosis. Concerning the *K*_*i*_ image, the original *K*_*i*_ image generated in the first 30 min was predicted accurately for the hypermetabolic region because the TAC of the hypermetabolic region showed an upward trend in the early stage and quickly entered the linear stage on the Patlak plot. The *K*_*i*_ image acquired by our method presents the same conclusions as the SUV forecast.

### Quantitative image quality assessment

**Fig. 7:**
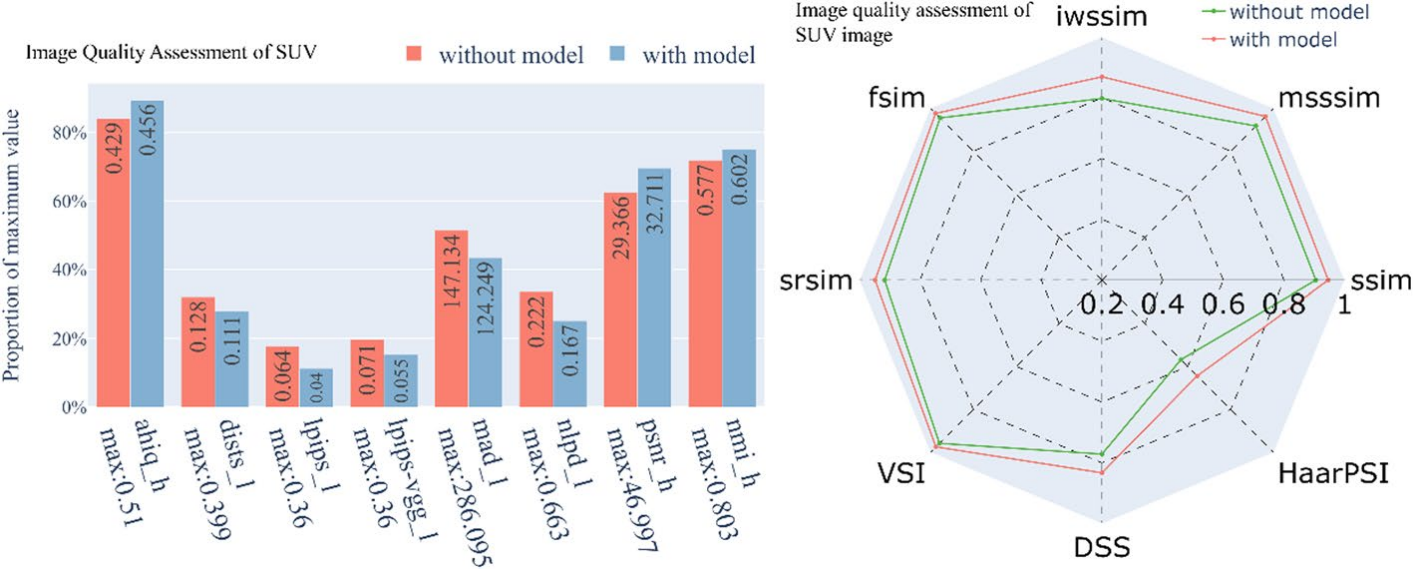
A comparison of the image quality of the SUV images produced with and without a kinetic model, where a real 1-h static PET image was used as a reference. The suffix “_h” means that the higher this metric is, the better the image quality. In contrast, the suffix “_l” means that this metric is a distortion index, so the lower this metric is, the better the image quality. The assessment of the radar image on the right shows that the larger the footprint is, the better the image quality

**Fig. 8:**
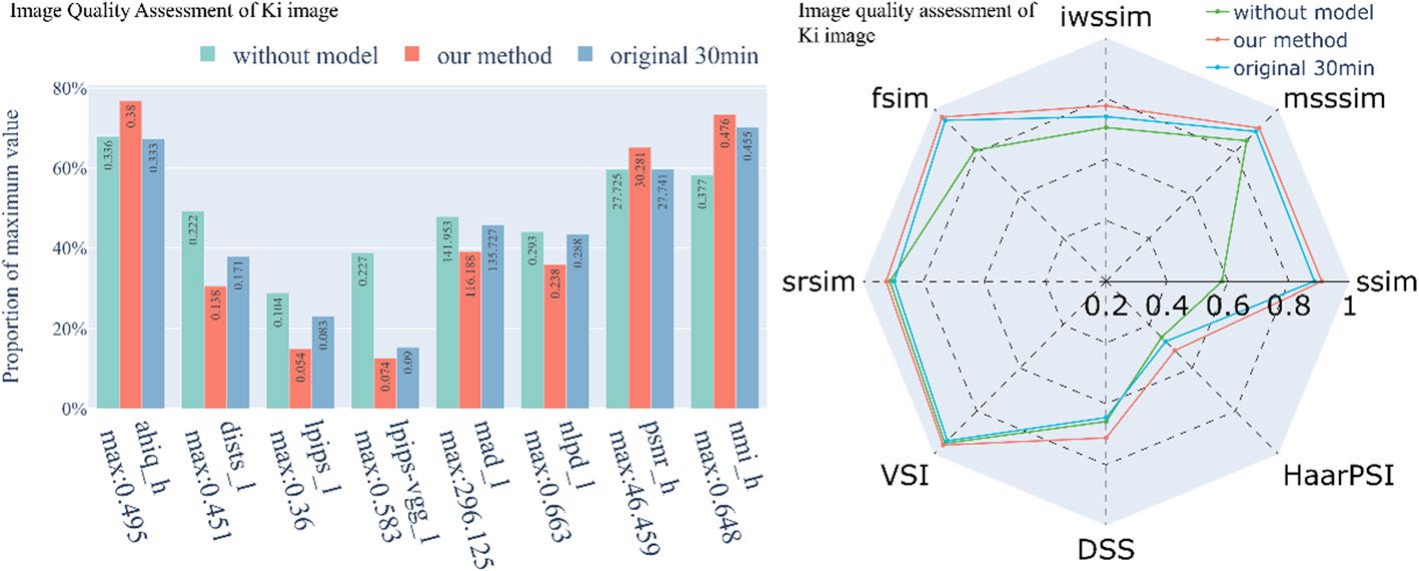
Image quality assessment of the *K*_*i*_ images generated by different methods with the same input. The explanations and descriptions of the pictures are the same as those in Fig. 7

We compared the image evaluation metrics computed by various deep learning methods, such as the attention-based hybrid image quality (AHIQ) method [23], the deep image structure and texture similarity (DISTS) approach [24], and the learned perceptual image patch similarity (LPIPS) technique [25], and some metrics without deep learning, such as gradient magnitude similarity deviation (GMSD) [26], most apparent distortion (MAD) [27], the normalized Laplacian pyramid distance (NLPD) [28], and the visual saliency-induced index (VSI) [29]. We also included traditional metrics such as the structural similarity index measure (SSIM), the peak SNR (PSNR), the normalized mutual information (NMI), and their improved versions such as the multiscale SSIM (MS-SSIM) [30], information content-weighted SSIM (IW-SSIM) [31], feature similarity index measure (FSIM) [32], spectral residual-based similarity index measure (SR-SIM) [33], discrete cosine transform (DCT) subband similarity (DSS) [34], and Haar perceptual similarity index (HaarPSI) [35] (Figs. 7, 8). These measurement methods showed that, on average, our method worked better than the *K*_*i*_ images made from 30 min of dynamic PET images.

**Fig. 9:**
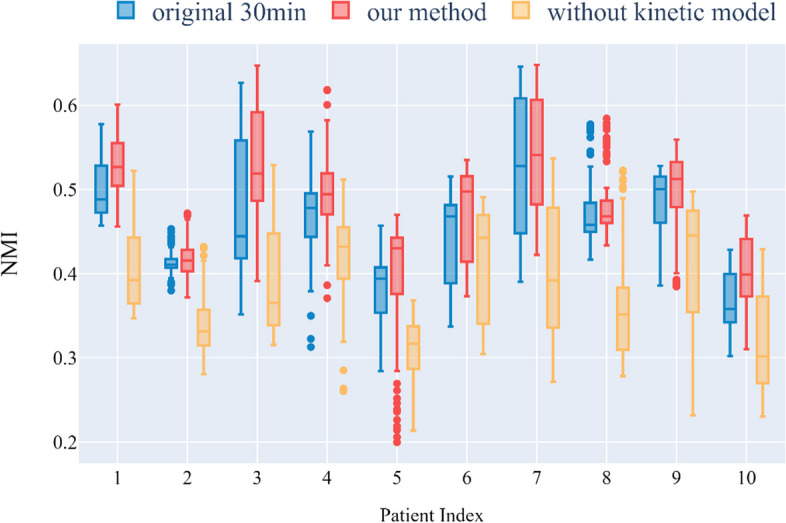
Comparison of the NMI distributions obtained with different patients and different methods

**Fig. 10:**
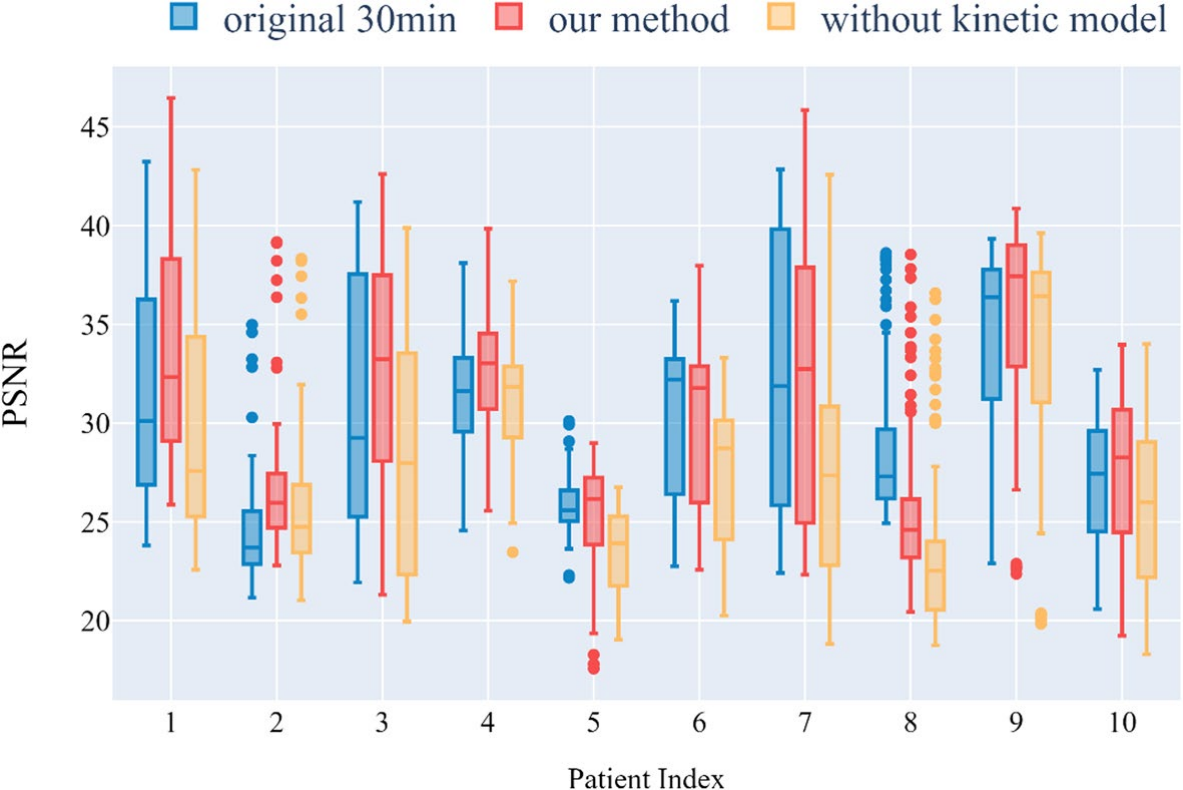
Comparison of the PSNR distribution obtained with different patients and different methods

**Fig. 11:**
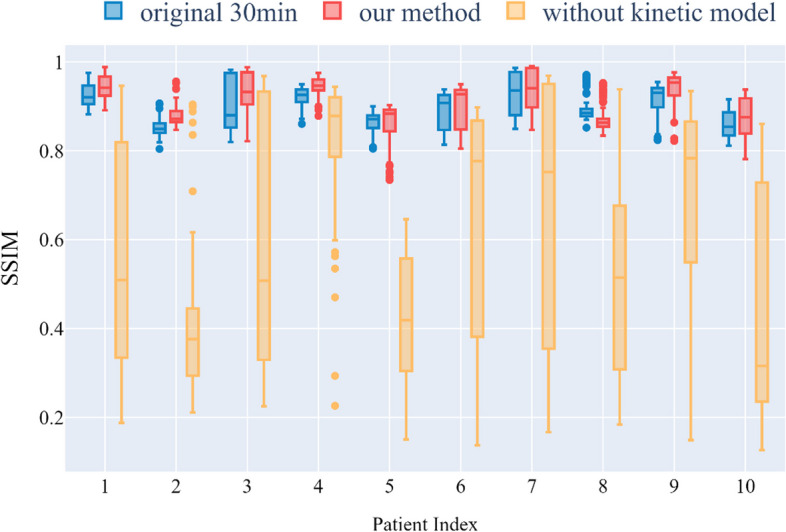
Comparison of the SSIM distributions obtained with different patients and different methods

Figure 9 shows that our method produced consistently better NMI metrics for all 10 patients' data when the ground truth was used as the reference image. This goes some way toward explaining the usability of our approach. Figure 10 shows that, except for the eighth patient, our method yielded better PSNR measurements than the original method. Figure 11 shows that the SSIM decreased significantly if the parameter image was made directly without using a kinetic model. However, this problem did not occur with our proposed method, and it can be seen that our method obtained better SSIMs for all patients except for patient 8.

To determine how close the synthetic *K*_*i*_ images were to the real images, a test subject with a malignant lung tumor was chosen from the test data. The region of interest (10 × 10 × 10) of the subject's tumor was delineated and analyzed in a Bland‒Altman plot. The Bland‒Altman plots showed that the 95% limits of agreement between the ground truths and the *K*_*i*_ images synthesized by the algorithm in this paper were between -0.029 ~ 0.03 (mean: 0.00), and the 95% limits of agreement between the ground truths and this *K*_*i*_ images synthesized by the method without incorporating the kinetic model were between − 0.027 and 0.034 (mean: 0.003), which were slightly larger than those of our proposed network. The 95% limits of agreement between the *K*_*i*_ images generated only with the original data acquired in the first 30 min and the ground truths were between − 0.029 and 0.039 (mean: 0.005), presenting the largest error.

## Discussion

We developed a new way to quickly and effectively combine deep learning with kinetic models to form dynamic PET images for the next 30 min from the dynamic PET images of the first 30 min. This method is the first time that SUV and *K*_*i*_ parametric images have been made at the same time, and it works well. Real patient data were used to show that the proposed method can make parametric images that match the reference images derived from Patlak plots. By using different metrics, such as evaluation criteria involving deep learning and metrics using the traditional computational method of extracting texture features for evaluating image quality, we showed that the image quality generated by our deep learning method combined with a kinetic model is better for *K*_*i*_ parameter images. This development may significantly reduce the required scanning time and improve patient comfort.

According to our observations, the SUV images generated by our method contained a certain amount of dynamic PET trend information for the first 30 min while bound by the curve of the kinetic model. If the target tissue's TAC does not fit the current kinetic model, it will not be suitable for constructing highly accurate parametric and SUV images. Additionally, because the generated images are learned from the input of the dynamic PET SUV source, if the input source does not contain the trend of the next 30 min, then the images will not be generated well either.

The *K*_*i*_ images generated by directly using a deep learning approach cannot guarantee consistency with the real situation, which can be seen in the SSIM metric comparison (Fig. 10), and the interpretability of deep learning is very low, which limits the application of deep learning in the medical field. Our method uses a kinetic model to make deep learning more interpretable to a certain degree.

The deep learning framework we proposed is also scalable. In future, as the level of pharmacokinetic modeling of human tissues and our understanding of how human tissues work metabolically improve, the TACs made with our method will become more accurate.

## Conclusion

In this work, we looked at an approach that combines kinetic models with deep learning using only the first 30 min of dynamic PET images to obtain the next 30 min of dynamic PET images and parametric *K*_*i*_ images. On data acquired from 103 patients, deep learning techniques combined with kinetic models were evaluated in terms of subjective and objective measures. The results showed that accurate parametric *K*_*i*_ image estimation is valid, can reduce the required scanning time and can make patients more comfortable. Although the proposed method performed well in quantitative evaluations, further validation is needed in clinical applications. In future, more research should be done on the kinetic modeling process to improve the performance of the existing models. For example, pharmacokinetic models that work for both tumors and normal tissues could be studied to make neural network models much more accurate.

## Data Availability

The datasets used or analyzed during the current study are available from the corresponding author upon reasonable request.

## Abbreviations

PET: Positron emission tomography
SUV: Standard uptake value
SNR: Signal-to-noise ratio
TAC: Time activity curve
SSIM: Structural similarity index measure
PSNR: Peak signal-to-noise ratio
RMSE: Root mean square error
FDG: Fluorodeoxyglucose
ADMM: Alternating direction of multipliers method
ReLU: Rectified linear unit
ODE: Ordinary differential equation
2TCM: 2-Tissue compartment model
Adam: Adaptive moment estimation
AHIQ: Attention-based hybrid image quality
DISTS: Deep image structure and texture similarity
LPIPS: Learned perceptual image patch similarity
GMSD: Gradient magnitude similarity deviation
VSI: Visual saliency-induced index
MAD: Most apparent distortion index
NLPD: Normalized Laplacian pyramid distance
NMI: Normalized mutual information
MS-SSIM: Multiscale SSIM
IW-SSIM: Information content-weighted SSIM
FSIM: Feature similarity index measure
SR-SIM: Spectral residual-based similarity index measure
DCT: Discrete cosine transform
DSS: DCT subband similarity
HaarPSI: Haar perceptual similarity index
